# The Week's Work

**Published:** 1911-09-30

**Authors:** 


### The Week's Work.

### SUNDAY CINEMATOGRAPHS.

The criticisms which were forcibly urged by members of the Council of the Hospital Sunday Fund against cinematograph exhibitions and their probable effects both upon the Sunday collections and upon the revenue of the two or three hospitals which have favoured them, are justified by the report of a meeting of the City of London Guardians on September 19. At that meeting, on the motion of Mr. Marr, it was unanimously resolved that the usual recommendation to give an annual donation of £21 to the London Hospital be referred back to the Finance Committee. The speeches show that the objections were based on the opinion that the London Hospital had been guilty of "injudicious action" and "had done a great deal to impair the success of the Hospital Sunday Fund." Mr. Marr stated that other hospitals had come into line and would not take cinematograph money in future. The effect of the resolution is that this subscription will be withheld from the London Hospital until the Guardians have an assurance that money will no longer be received by that institution from Sunday cinematograph shows. The importance of this decision is emphasised by the large falling off which has taken place in the total sum received by the Sunday Fund from church collections this year.

### THE MANCHESTER CONFERENCE.

As we go to press we understand that there is every indication that the British Hospitals Association Conference, which takes place at Manchester this week, will be a highly successful gathering. Hospital workers may reasonably look forward to a full discussion of the several important problems of hospital politics at this meeting which will focus public attention upon the outstanding features of interest in the institutional world. We have arranged to report the most important discussions in full, and in next week's issue of The Hospital we hope to give a detailed account of the Congress, together with abstracts of the papers read.

### HOSPITAL PROGRESS ON THE GOLD COAST.

This African colony is associated so much in the public mind with disease, death, and disaster, that it is well to notice the encouraging development of its hospitals and dispensaries. The Governor of the Gold Coast, in his annual Blue-book, has some interesting statistics on the point. There are eleven hospitals in the colony, and during the year new hospitals were completed at Tarquah, Akuse, and Winnebah, and a special fly-proof hospital was built at Anum, in the Volta District, for the treatment of sleeping sickness, and isolation and observation of suspected cases. Altogether no fewer than 900 Europeans and over 26,000 natives received treatment. In Ashanti there is, at Coomassie, a European hospital with four wards. There is also a well-equipped native hospital, containing three wards. In the Northern Territories, at Tamale, a new hospital is now being built. In Accra the hospital consists of three European and four native wards, containing seven and twenty-five beds respectively. At Cape Coast the hospital contains accommodation for thirteen Europeans, in three first-class and two second-class wards. We thus note the beginnings both of racial and economic classification. The former is recognised in London by the Hebrew wards which more than one London hospital possesses. Economic classification, on a modified form of the American graduated pay wards, has yet to be undertaken in the old country. The total number of patients admitted to the mental hospital during the year was forty-four males and six females, who are chiefly employed in gardening. The mental hospital, which is situated at Accra, consists of two large airing yards, one for males and the other for females, each flanked by dormitories. There is also a large open shed for shelter and recreation. Hospital accommodation, quarters for the staff and other accessories, are provided, and the buildings generally are, we believe, well-ventilated and cool.

### NEW CHAIRMAN FOR THE COVENTRY HOSPITAL

Mr. Alfred Herbert has felt it his duty to maintain his original decision, and so with the most genuine regret the Board of the Coventry and Warwickshire Hospital has been compelled to accept his resignation. His services to the institution were made the subject of generous comment in the Report of the hospital, which we published on December 25, 1909. On Wednesday last week the General Committee met, the election of a new chairman being placed on the agenda. Mr. Edward Iliffe, who has filled the post of vice-chairman for the past two years, was then elected chairman for the remainder of the year. The election of the new vice-chairman will be made shortly.

### QUEER DEFINITIONS IN HOSPITAL DIET.

The question, should a hospital provide free treatment only, or should it also provide all food free, still excites discussion. The most interesting point, perhaps, is the origin of the practice, for the practice itself, as Mr. Morris has pointed out, can find very able defenders. Mr. Morris supplies various reasons why tea, sugar, and butter should be charged for at the present time. He does not, however, prove, in our opinion, that these arguments were the governing considerations originally. He explains that tea, sugar, and butter may be charged for now because patients are relatively better off than they were "when most of our hospitals were founded." This, however, implies that formerly patients were poorer, and as such could not be expected to pay. Why, then, were they expected to do so? It is here that Mr. Thies' argument has weight. In our opinion tea, sugar, and butter have been charged for at the London and at many other hospitals for different reasons at different times. Originally, we fancy, with Mr. Thies, they were charged for because they were regarded as luxuries. Now, as Mr. Morris shows, they are charged for as being, like a few articles of linen, simple necessities for which the modern class of patient may be expected reasonably to pay. Mr. Morris, we would suggest, is possibly mistaken in saying that they have always been charged for for the same reason. Mr. Thies, we think, is in error in claiming that the original reason is still valid. We have thought it more useful to disentangle the threads of this particular discussion than to repeat here the facts and opinions which formed the subject of a special chapter in "Burdett's Hospitals and Charities" for 1893. The burning questions are always the old ones. Pythagoras and Plato are always topical.

### THE LATE MR. W. G. BUNN.

We publish elsewhere a brief memoir of the work and remarkable personality of Mr. W. G. Bunn, who for seventeen years acted as Secretary of the Hospital Saturday Fund. Only three years ago reasons of health compelled him to resign this post, and it is noteworthy that the Hospital Saturday Fund was only the centre of a wide range of interests, which included mutual benefit societies of various descriptions, in the historical aspects no less than in the functions of which he took a very keen interest. The funeral took place last Monday at Golders Green, for Mr. Bunn had expressed a wish to be cremated, and at the funeral the Hospital Saturday Fund was represented, among others, by Mr. A. W. Davis, Mr. Bunn's successor in the secretaryship.

### THIS WEEK'S DRUG MARKET.

The demand for drugs has been rather more active, and it is not unlikely that as the winter season approaches a considerable improvement in business will be noticed in the drug market. The most important change in value is in the case of santonin, the price of which has been advanced by 5s. per lb.; the alteration in price was not altogether unexpected, and as the drug is controlled by a syndicate there is no limit to the price to which it may be raised. Its present value is something like ten times the figure which was quoted at one time. There is no change in the position of opium, morphine, and codeine, but prices are well maintained. The demand for cod-liver oil may be expected to set in shortly, and at present there are no signs of a tendency for prices to give way. Oil of aniseed is rather dearer, and a fair amount of business has been done. Other articles which have advanced in price to a small extent are castor oil, isinglass, carbolic acid, and cantharides. On the other hand, ipecacuanha and myrrh are slightly lower in value. In the case of cream of tartar the tendency is towards lower quotations in view of the favourable reports from wine-growing districts.

